# Dragon Grab Bars and Bamboo Canes: Exploring How Older Chinese Perceive Home Modifications and Assistive Devices

**DOI:** 10.1093/geroni/igaf122.2826

**Published:** 2025-12-31

**Authors:** Mengzhao Yan, Maria Henke, Aaron Hagedorn, Li-Mei Chen, Jon Pynoos

**Affiliations:** University of Southern California, Los Angeles, California, United States; University of Southern California, Los Angeles, California, United States; University of Southern California, Los Angeles, California, United States; George Mason University, Fairfax, Virginia, United States; University of Southern California, Los Angeles, California, United States

## Abstract

Aging in place continues to be the preference for most people, but the majority of homes are not equipped to handle their evolving care needs. Although home modifications and assistive devices can enable people to stay in their homes, previous research indicates that minority groups, who often have a stronger inclination to age at home, are less likely to modify their home environment. This issue is often compounded by the shortage of culturally tailored products and services. To explore how perceptions of home modifications and assistive devices are relevant to cultural backgrounds, this pilot qualitative study focuses on older Chinese Americans. Using person-environment fit and cultural adaptation as a framework, we conducted seven semi-structured, in-depth interviews in 2024 with four older Chinese Americans (aged 70 to 95) who made home modifications or used assistive devices and three of their caregivers. Using thematic analysis to examine the interview notes, we identified four themes: (1) Helpfulness. Interviewees generally appreciated supportive features that improved daily living and independence; (2) Learning Curve. Both older adults and caregivers noted the efforts required to adapt to the changes; (3) OT Support. Older adults who made home modifications acknowledged the instrumental role of occupational therapists. (4) Cultural Influence. People prefer designs that reflect their culture. Our findings suggest that, while some aspects of home modifications and assistive devices are consistently valued among older minorities, an improved cultural competency in product design and service delivery can help create person-centered solutions.

